# Predictive value of postoperative prealbumin levels for detecting early-stage complications following off-pump coronary artery bypass grafting

**DOI:** 10.3389/fcvm.2025.1476053

**Published:** 2025-02-13

**Authors:** Yanting Jia, Jun Zhang, Lei Chen, Yanhui Zhu

**Affiliations:** Department of Cardiac Surgery, Shandong Provincial Hospital Affiliated to Shandong First Medical University, Jinan, Shandong, China

**Keywords:** atrial fibrillation, complications, inflammatory response, off-pump coronary artery bypass grafting, prealbumin

## Abstract

**Background:**

A pathological decrease in the serum prealbumin level is closely associated with the severity of various diseases and complications after surgery. Many patients suffer from a systemic inflammatory response and local myocardial ischemia after off-pump coronary artery bypass grafting, which is related to an adverse prognosis. This study aimed to explore the relationship between the serum prealbumin level and early complications following off-pump coronary artery bypass grafting, in addition to its predictive value.

**Methods:**

Data were retrospectively collected on patients undergoing off-pump coronary artery bypass grafting from January 2014 to July 2019. The serum prealbumin level was assessed within 6–12 h after the operation. Patients were classified into two groups: the “normal” level group (≥17 mg/dl) and the “low” level group (<17 mg/dl). Univariate and multivariable analyses were performed to evaluate the predictive value of a low serum prealbumin level.

**Results:**

Of the 1,002 patients, 553 (55.2%) had a low serum prealbumin level. The rate of pulmonary infection in the low group was significantly higher than in the normal group. Univariate analysis and multivariable analysis indicated that a low serum prealbumin level was associated with the increased incidence of postoperative pulmonary infection, pleural effusion, and new-onset atrial fibrillation.

**Conclusion:**

The serum prealbumin level following off-pump coronary artery bypass grafting serves as a crucial predictor for early major postoperative complications, such as pulmonary complications and new-onset atrial fibrillation. Early identification of high-risk patients by assessing serum prealbumin levels, in addition to the timely adjustment of treatment and care strategies, can improve patient prognosis, shorten hospital stays, and reduce healthcare expenses.

## 1 Introduction

It has recently been revealed that in pathological states, such as inflammation and stress, reduced serum prealbumin (PA) levels are closely associated with the severity of several diseases and postoperative complications, and can even serve as a vital indicator for the stratification of surgically critically ill patients (1–6). Therefore, PA is not only a nutritional indicator, but also acts as an independent risk factor in certain pathological states, predicting or even influencing the disease regression. However, in the cardiovascular field, few reports on the relationship between PA levels and disease progression, particularly its role in the postoperative assessment following cardiac surgery, are available.

Therefore, this large-sample, retrospective study aimed to investigate the relationship between the PA level and early-stage complications following off-pump coronary artery bypass grafting (OPCAB), in addition to its predictive value. These findings may help to guide perioperative treatment and promptly adjust care strategies to help patients' postoperative recovery, which has great clinical significance.

## 2 Materials and methods

Data were collected retrospectively from 1,485 patients who had undergone OPCAB surgery at a single institution between June 2014 and July 2019. The study was approved by the Institutional Review Board of our hospital. The data are anonymous, and the requirement for informed consent was therefore waived.

Inclusion criteria for patients were as follows. (1) Age >18 years old. (2) Isolated first-time OPCAB surgery. (3) Normal liver and kidney function before operation.

**Figure 1:**
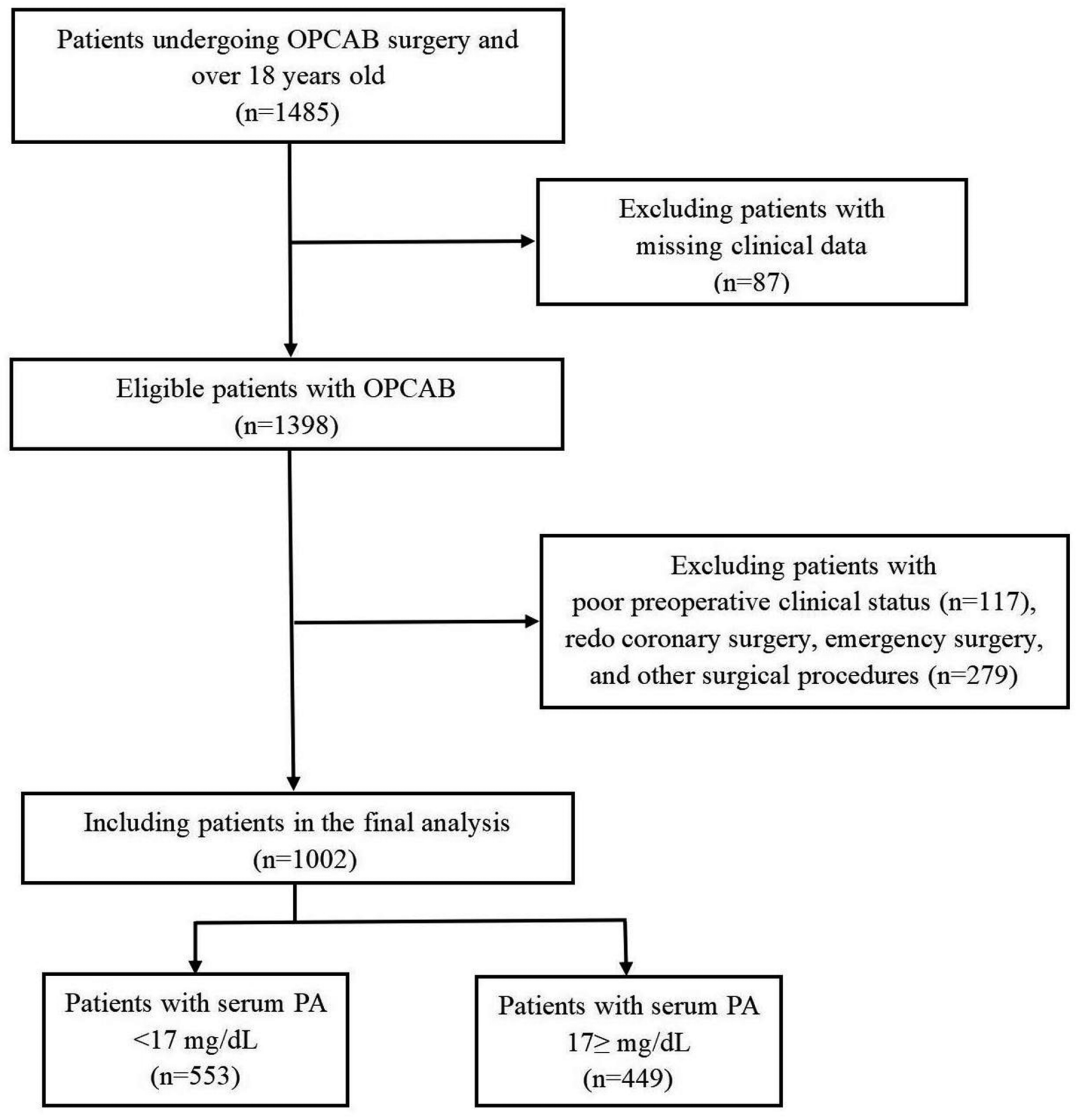
Flowchart depicting patients selection in the study. OPCAB, off-pump coronary artery bypass grafting; PA, prealbumin.

The exclusion criteria were as follows: (1) Missing clinical data (*n* = 87). (2) Poor preoperative clinical status (e.g., hepatic or renal insufficiency, malignancies with low life expectancy, chronic inflammatory disease, active infection, preoperative inotropic agents and/or intra-aortic balloon pump (IABP) requirement, history of cardiopulmonary resuscitation within last 4 weeks) (*n* = 117). (3) Redo coronary surgery, emergency surgery, and concomitant valve surgery, left ventricular surgery, or other surgical procedures than isolated OPCAB (total *n* = 279). The remaining 1,002 patients were include in our final analysis (Figure 1).

### 2.1 Observational indicators

Fasting venous blood samples were collected from all patients within 6–12 h after surgery. Data on the patient's characteristics were collected retrospectively, and preoperative demographics and clinical features, intraoperative data, and postoperative outcomes were all recorded and then analyzed. In the present study, we have used PA 17 mg/dl as the cut-off point, which is the most widely reported value used in the literature (7–10). Accordingly, patients were divided into two groups: serum PA <17 mg/dl (“low” level group) and serum PA ≥17 mg/dl (“normal” level group).

Clinical and procedural data were recorded retrospectively on standardized forms by trained medical personnel. Medical records were reviewed to obtain information regarding patient demographics and comorbidities, operative techniques, postoperative management, complications, and outcomes. The primary outcomes of this study were as follows: extubation time; intensive care unit (ICU) length of stay; postoperative hospital stay; the necessity of inotropic support and IABP; postoperative complications including pulmonary infection, pleural effusion, new-onset atrial fibrillation (AF), hepatic dysfunction, cerebrovascular event (CVE), incision problem, respiratory failure, perioperative myocardial infarction (MI), low left ventricular ejection fraction (LVEF), and acute renal failure (ARF) requiring hemodialysis; and in-hospital mortality.

### 2.2 Definitions

Pulmonary infection was defined as the presence of purulent sputum associated with fever or requiring antibiotic therapy according to a positive sputum culture. Pleural effusion was defined as middle-to-large pleural effusion confirmed by ultrasound or x-ray. New-onset AF was defined as a new-onset AF during the postoperative hospitalization that required treatment and was diagnosed based on 12-lead ECG findings. Hepatic dysfunction was an elevated postoperative alanine transaminase (ALT) level >100 U/L or an elevated serum bilirubin level >3 mg/dl.

CVE was confirmed by computed tomography and a neurologic consultation. An incision problem was defined as any impaired wound healing, including superficial and deep wound infection. Respiratory failure was defined as the requirement for the continuation of mechanical ventilation beyond 48 h after surgery. A diagnosis of perioperative MI was confirmed by the following events: an elevation in the plasma level of cardiac troponin I (cTnI) >10 times the upper limit of normal in addition to the development of new pathological Q waves or echocardiographic evidence of new-onset regional left ventricular wall motion abnormalities. A low LVEF was defined as <50%. ARF was defined as the requirement for renal replacement therapy after the operation. Hospital mortality was defined as any-cause mortality in the hospital after the surgery.

### 2.3 Statistical analysis

Preoperative, operative, and postoperative variables were compared between the two groups by adopting different statistical tests. Categorical variables were analyzed using the Chi-square test or Fisher's exact test. For the analyses of normally distributed continuous variables, independent samples *t*-tests were used. The nonparametric Mann–Whitney test was used for nonnormal data. While continuous variables were expressed as median with interquartile ranges, categorical variables were presented as frequency and percentage for each group. Correlations between continuous variables were determined using Spearman's correlation coefficient.

Univariate logistic regression was used to analyze the prognosis-related factors; those with predictive value were subjected to the multivariate logistic regression analysis. Factors with confounding effects or interactions were excluded to clarify the predictive value of PA. Subsequently, the screened independent predictors were used to plot the receiver operating characteristic (ROC) curves and calculate the area under the curve (AUC). Finally, an AUC comparison between the two groups was performed by the *Z*-test to evaluate the predictive performance of PA.

All statistical analyses were performed using GraphPad Prism 9.0 (© GraphPad Software, Inc., La Jolla, CA, USA). In addition, the statistical tests were two-sided with a *P*-value <0.05 considered statistically significant.

## 3 Results

Of the 1,002 patients, 553 (55.2%) had a PA level <17 mg/dl. There were no differences in demographics, comorbidities, clinical characteristics, echocardiographic data, and surgical data between the two groups (Table 1). The correlation analysis indicated extremely weak correlations between the postoperative levels of PA and the preoperative levels of albumin (*r* = 0.117, *P* = 0.0002) and body mass index (*r* = 0.0665, *P* = 0.0352).

**Table 1:** Baseline demographic and clinical characteristics between the normal and low postoperative serum prealbumin groups.

Baseline demographic and clinical characteristics	Postoperative prealbumin		*P*-value
	≥17 mg/dl (*n* = 449)	<17 mg/dl (*n* = 553)	
Demographics			
Age, year	63 (57.7–68)	63 (58.4–68.1)	0.176
Female, *n* (%)	118 (26.28%)	172 (31.10%)	0.094
Clinical characteristics			
BMI, kg/m^2^	25.3 (23.5–27.7)	24.9 (23.0–27.3)	0.071
BMI <20, *n*	16	20	0.964
BMI 20–30, *n*	410	492	0.218
BMI >30, *n*	23	41	0.14
EuroSCORE II	1.1 (0.88–1.34)	1.14 (1.88–1.48)	0.097
Laboratory and echocardiographic data			
Preoperative albumin, g/L	42.1 (40–44.2)	40.9 (39–43.5)	<0.001
Preoperative SCr, μmmol/L	68 (62–78)	71 (62–78)	0.1213
LVEF (%)	60 (58–61)	60 (57–61)	0.164
Comorbidities			
Diabetes mellitus (%)	142 (31.63%)	198 (35.8%)	0.165
Hypertension (%)	289 (64.37%)	328 (59.31%)	0.102
Chronic lung disease (%)	9 (2.00%)	16 (2.89%)	0.37
Cerebrovascular disease (%)	59 (13.14%)	82 (14.83%)	0.445
Previous myocardial infarction (%)	119 (26.50%)	138 (24.95%)	0.577
Grafts per patient	3 (3–3)	3 (3–4)	0.071
Operation time, h	3.3 (3–3.5)	3.4 (3–3.6)	0.062

BMI, body mass index; EuroSCORE, European system for cardiac operative risk evaluation; SCr, serum creatinine; LVEF, left ventricular ejection fraction.

In the low-level group, the ICU length of stay, length of postoperative hospital stay, and extubation time were significantly longer, and the proportion of inotropic support, pulmonary infection, pleural effusion, new-onset AF, incision problems, and respiratory failure were higher than those in the normal-level group. However, there was no significant difference between the two groups regarding perioperative MI, hepatic dysfunction, CVE, low LVEF, necessity of IABP, ARF requiring hemodialysis, and in-hospital mortality (Table 2).

**Table 2:** Univariable analysis of postoperative prealbumin and postoperative outcome.

Characteristic	Postoperative prealbumin		*P*-value
	≥17 mg/dl (*n* = 449)	<17 mg/dl (*n* = 553)	
Necessity of inotropic support	39 (8.69%)	149 (26.94%)	<0.0001
Extubation time, h	7 (6–8)	8 (6.7–11.5)	<0.0001
ICU length of stay, days	1.9 (1.8–2.8)	2.1 (1.9–2.9)	<0.0001
Postoperative hospital stay, days	9 (8–10)	9 (8–11)	<0.0001
Neutrophils after surgery, ×10^9^/L	12.78 (10.87–14.79)	12.39 (10.17–14.75)	0.02
Postoperative complications			
Pulmonary infection	32 (7.13%)	161 (29.11%)	<0.0001
Pleural effusion	11 (2.45%)	89 (16.09%)	<0.0001
New-onset atrial fibrillation	39 (8.69%)	132 (23.87%)	<0.0001
Hepatic dysfunction	29 (6.46%)	34 (6.15%)	0.84
CVE	5 (1.11%)	10 (1.81%)	0.368
Incision problem/Wound infection	1 (0.22%)	13 (2.35%)	0.0098
Respiratory failure	2 (0.45%)	13 (2.35%)	0.027
Hospital mortality	2 (0.45%)	3 (0.54%)	>1
Necessity of IABP	0	1 (0.18%)	>1
ARF	1 (0.22%)	2 (0.36%)	>1
Perioperative MI	10 (2.23%)	22 (3.98%)	0.117
Low LVEF	27 (6.01%)	32 (5.79%)	0.88
MACE	37 (8.2%)	55 (9.9%)	0.353

ICU, intensive care unit; CVE, cerebrovascular event; ARF, acute renal failure; LVEF, left ventricular ejection fraction; MACE, major adverse cardiovascular events.

The univariate analysis of the postoperative PA levels and postoperative pulmonary infections revealed that low PA levels were associated with an increased incidence of pulmonary infections (*P* < 0.0001). Other risk factors for pulmonary infections were female sex, concurrent chronic lung disease, older age, longer surgery duration, longer extubation time, higher neutrophil count, and lower albumin level.

**Figure 2:**
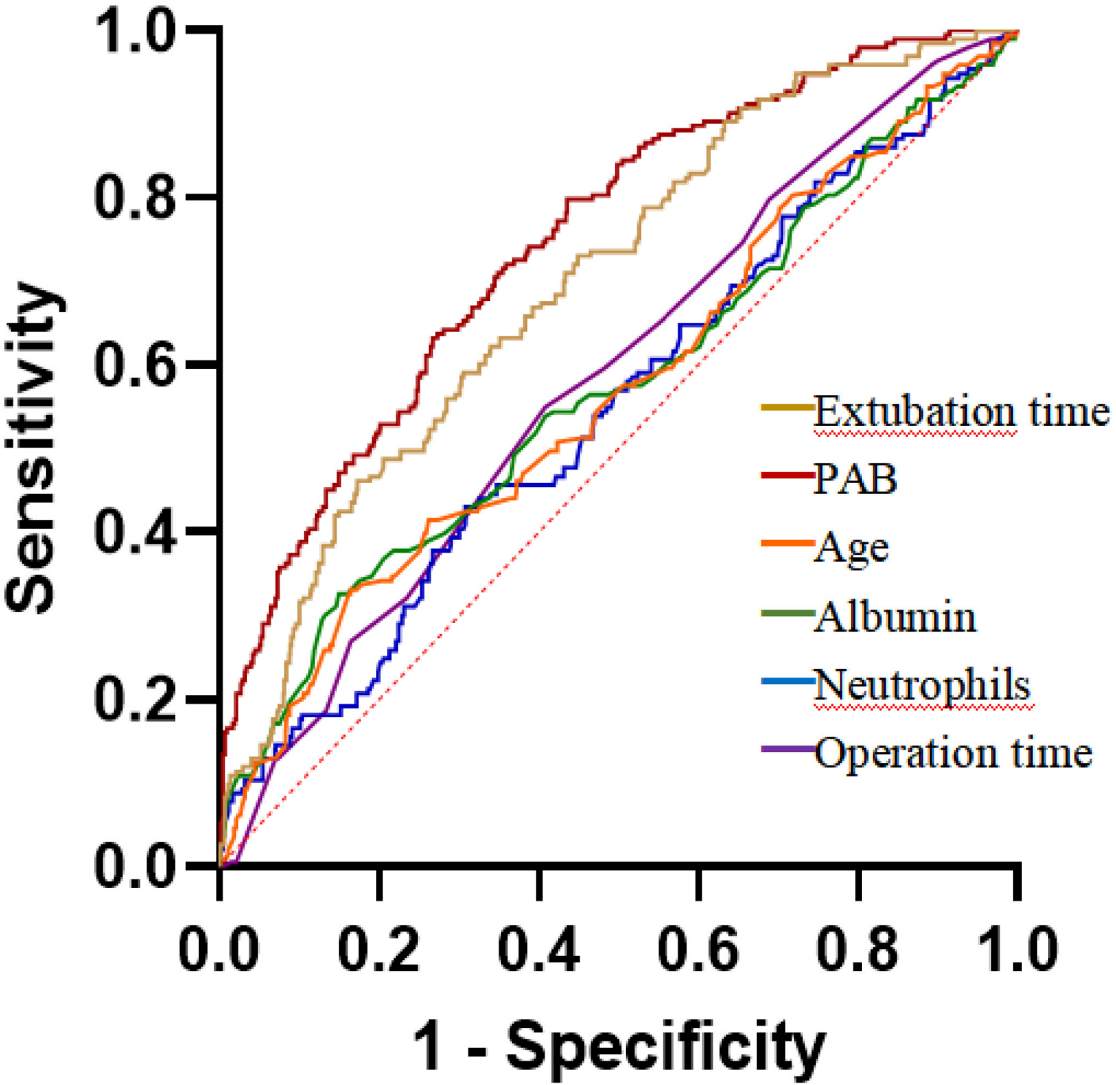
Receiver operating characteristics curves of PA and other variables in predicting postoperative pulmonary infection. The area under the curve of the PA and extubation time is 0.742 (95% CI: 0.704–0.781) and 0.684 (95% CI: 0.648–0.730), respectively (*P* = 0.043).

Furthermore, we conducted a multivariate logistic regression analysis of the significant factors. The result suggested that a lower preoperative PA level was an independent risk factor for postoperative pulmonary infection [adjusted odds ratio (OR): 1.5, 95% confidence interval (CI): 1.324–1.704, *P* < 0.0001]. Other independent risk factors included older age, longer operation time, longer extubation time, higher neutrophil count, and lower albumin level (Table 3). According to the ROC curves in Figure 2 and calculations in Table 4, the predictive power of the postoperative PA level was significantly stronger than other variables and decreased PA levels had a greater predictive value for postoperative pulmonary infection.

**Table 3:** Predictors of respiratory tract infection: univariate and multivariate approaches.

Characteristics	OR (95% CI) univariate	*P*-value	OR (95% CI) multivariate	*P*-value
Prealbumin (per mg/L)	0.977 (0.971–0.982)	<0.0001	1.5 (1.324–1.704)	<0.0001
Albumin (per g/L)	0.916 (0.869–0.964)	0.0008	0.973 (0.887–0.989)	0.019
Neutrophils (per 10^9^/L)	1.07 (1.023–1.119)	0.0031	1.12 (1.066–1.177)	<0.0001
Preoperative LVEF (%)	1.006 (0.973–1.042)	0.729	–	–
Female sex	1.48 (1.039–2.091)	0.023	1.025 (0.689–1.510)	0.902
Age (per year)	1.035 (1.014–1.057)	0.0012	1.029 (1.006–1.052)	0.0124
BMI (per kg/m^2^)	0.961 (0.911–1.013)	0.145	–	–
Chronic lung disease	2.424 (1.012–5.466)	0.037	2.032 (0.773–5.054)	0.135
Diabetes mellitus	1.168 (0.840–1.616)	0.351	–	–
Operation time (per h)	1.976 (1.327–2.953)	0.0008	1.725 (1.114–2.679)	0.0147
Extubation time (per h)	1.13 (1.091–1.174)	<0.0001	1.11 (1.070–1.155)	<0.0001
Grafts per patient	0.908 (0.708–1.161)	0.442	–	–

OR, odds ratio; CI, confidence intervals; LVEF, left ventricular ejection fraction; BMI, body mass index.

**Table 4:** ROC analysis of predictors in predicting postoperative respiratory tract infection.

Variables	AUC	95% CI	*P*-value	Std. error	Cut-off	Sensitivity (%)	Specificity (%)
Prealbumin, mg/L	0.742	0.704–0.781	<0.0001	0.0197	<179.5	73.42	64.55
Extubation time, h	0.684*	0.648–0.729	<0.0001	0.0207	>8.9	58.55	72.56
Age, year	0.571**	0.524–0.618	0.0022	0.0239	>70	33.16	83.56
Albumin, g/L	0.574**	0.526–0.622	0.0014	0.0246	<39	36.79	83.19
Neutrophils, ×10^9^/L	0.551**	0.505–0.598	0.026	0.0236	>14.04	43.01	69.22
Operation time, h	0.585**	0.541–0.629	0.0002	0.0224	>3.5	54.92	59.21

The AUC between each variable and PAB was compared by *Z*-test.

PAB, prealbumin; ROC, receiver operating characteristic; AUC, area under the curve; CI, confidence intervals; Std. Error, Standard error.

* *P* < 0.05.

** *P* < 0.01.

## 4 Discussion

The inflammatory response following cardiac surgery is an integrated process that includes endothelial dysfunction, platelet activation, and activation of multiple complementary components. OPCAB avoids the exacerbating factors encountered in cardiopulmonary bypass (CPB); however, in previous studies, patients who underwent OPCAB still exhibited a widespread perioperative inflammatory response in the absence of CPB (11–22), which can influence the clinical outcomes, particularly early complications. Therefore, it is theoretically possible that some rapid-response inflammatory indicators are associated with the early prognosis of OPCAB patients.

PA is a transport protein synthesized by hepatocytes; it is more sensitive than albumin due to its short half-life, rapid renewal, and minimal influence on liver disease and blood product transfusions (23). Phillip et al. (24) induced an inflammatory response in rats by subcutaneous injection of turpentine and reported a faster and more significant decrease in PA than in albumin during the acute phase of inflammation. Recent research also shows that decreased PA levels during periods of the inflammatory response are often not the result of calorie or protein deficiency, but are negatively correlated with inflammatory biomarkers, indicating that they are likely to signal an inflammatory response and poor prognosis (25).

Based on previous studies (1–6), we selected the early postoperative PA level of OPCAB patients as an observation indicator to predict postoperative complications.

As an inflammatory response signal, PA is theoretically predictive of the frequency of postoperative pulmonary injuries. Pulmonary complications after cardiac surgery include atelectasis, pleural effusion, pneumonia, pulmonary edema, acute respiratory distress syndrome, pulmonary embolism, aerothorax, and sternal wound infections. Pulmonary complications are the most prevalent and unavoidable of all postoperative cardiac complications.

This study primarily includes postoperative pulmonary infection, pleural effusion, incision problems, and respiratory failure from any cause. These complications were significantly more prevalent in the low PA group than in the normal group. Pulmonary infection is the most common nosocomial infection in the postoperative period, with a reported incidence of postoperative pneumonia ranging from 6.37% to 35.2% in high-risk groups and a mortality rate of up to 42% at 30 days postoperatively (26, 27). We discovered a statistically significant difference in the incidence of postoperative lung infection in the low PA group. Subsequently, postoperative pulmonary infection was made the study subject, and we introduced numerous potentially significant factors for correction analyses. The results demonstrated that decreased postoperative PA, advanced age, prolonged operative time, prolonged extubation time, elevated postoperative neutrophils, and decreased albumin levels were independent risk factors for postoperative pulmonary infections. Some previously mentioned significant factors, such as gender, diabetes, and comorbid chronic pulmonary diseases, were excluded, probably due to differences in care strategies. Furthermore, due to the limitations of retrospective research, not all influencing factors have been validated (Table 3).

Using ROC curve analysis, we discovered that PA and extubation time had the highest AUCs of 0.742 and 0.684, respectively, with ideal cut-off values of PA <179.5 g/L and extubation time >8.9 h, respectively, while the other predictors are less effective. When compared by the *Z*-test, PA is the strongest predictor of postoperative pulmonary infection, and we found that by combining PA and extubation time in a ROC curve (AUC = 0.807), the predictive efficacy is further improved, which was significantly higher than the individual index (Table 4, Figure 2). In previous studies, many risk factors for postoperative infectious complications after coronary artery bypass grafting have been identified. The importance of our study is that we have shown, for the first time, that PA is an independent predictor of postoperative pulmonary infection after OPCAB, and its predictive efficacy is significantly higher than other variables, signifying that PA levels <179.5 g/L result in a greater potential for early infection.

AF is one of the most common cardiac arrhythmias after cardiac surgery, and its prevalence is rising with the aging population. Despite advances in anesthesia and surgical techniques, which have reduced the risk of postoperative complications in patients undergoing cardiac surgery, AF remains the most common complication, with a prevalence ranging between 10% and 50%. However, the mechanism for AF after cardiac surgery is uncertain, and inflammation is assumed to be the primary pathophysiology (28–31).

In our study, PA was found to be closely associated with the incidence of new-onset postoperative AF in OPCAB patients as an indicator of inflammation, with a considerably higher incidence of AF in the low PA group than in the normal group (Table 2). However, regression analysis and the ROC curve show that although the PA level is an independent influential factor for new-onset postoperative AF after surgery, the prediction efficacy is not high. Therefore, new-onset postoperative AF after OPCAB may be influenced by a combination of the perioperative inflammatory response and preoperative high-risk factors. Furthermore, the mechanism may be complex, restricting its ability to be predicted by a single factor.

This study has several limitations that are worth noting. First, it is a single center, retrospective, non-randomized controlled study. The acquisition of statistical data has limitations and is unable to obtain all potentially meaningful indicators for the analysis. Therefore, the retrospective nature of this study could have inevitably contained a certain level of selection bias. Second, due to the limitation of the research method, PA was measured after surgery; the blood collection time was within 6–12 h after surgery, not at the same time. As a result, the data lacks certain homogeneity. Third, the division of normal PA and low PA groups is based on the common criteria used in previous studies, which may have some influence on the differential analysis results of some prognosis indicators. Fourth, only short-term studies of prognosis indicators during the postoperative hospitalization period were conducted, with no long-term follow-up; therefore, the impact of changes in the PA level on the medium and long-term prognosis after OPCAB is unclear. Last, on-pump cases means that there will be more influencing factors during the surgical process, so our results may not be fully applicable to on pump cases. As a result, the specific influencing factors and the role of PA may necessitate the accumulation of more cases and further classification studies.

## 5 Conclusion

In conclusion, the PA level after OPCAB serves as a crucial predictor for early major postoperative complications, such as pulmonary complications and new-onset AF. The early identification of high-risk patients by PA levels, in addition to the timely adjustment of treatment and care strategies, can improve patient prognosis, shorten the hospital stay, and reduce healthcare expenses. Further studies are needed to better determine the effect of PA on the long-term prognosis of OPCAB patients in the future.

## Data Availability

The raw data supporting the conclusions of this article will be made available by the authors, without undue reservation.
